# The Tumor Immune Microenvironment and Frameshift Neoantigen Load Determine Response to PD-L1 Blockade in Extensive-Stage SCLC

**DOI:** 10.1016/j.jtocrr.2022.100373

**Published:** 2022-07-01

**Authors:** Hiroaki Kanemura, Hidetoshi Hayashi, Shuta Tomida, Junko Tanizaki, Shinichiro Suzuki, Yusuke Kawanaka, Asuka Tsuya, Yasushi Fukuda, Hiroyasu Kaneda, Keita Kudo, Takayuki Takahama, Ryosuke Imai, Koji Haratani, Yasutaka Chiba, Tomoyuki Otani, Akihiko Ito, Kazuko Sakai, Kazuto Nishio, Kazuhiko Nakagawa

**Affiliations:** a Department of Medical Oncology, Kindai University Faculty of Medicine, Osaka, Japan; b Center for Comprehensive Genomic Medicine, Okayama University Hospital, Okayama, Japan; c Department of Medical Oncology, Kishiwada City Hospital, Osaka, Japan; d Department of Medical Oncology, Izumi City General Hospital, Osaka, Japan; e Department of Respiratory Medicine, Kurashiki Central Hospital, Okayama, Japan; f Department of Clinical Oncology, Graduate School of Medicine, Osaka City University, Osaka, Japan; g Department of Thoracic Medical Oncology, National Hospital Organization Osaka Minami Medical Center, Osaka, Japan; h Department of Medical Oncology, Kindai University Nara Hospital, Nara, Japan; i Department of Pulmonary Medicine, Thoracic Center, St. Luke’s International Hospital, Tokyo, Japan; j Clinical Research Center, Kindai University Hospital, Osaka, Japan; k Department of Pathology, Kindai University Faculty of Medicine, Osaka, Japan; l Department of Genome Biology, Kindai University Faculty of Medicine, Osaka, Japan

**Keywords:** Small cell lung cancer, Immunotherapy, Tumor-infiltrating lymphocyte, Tumor mutation burden, Neoantigen

## Abstract

**Introduction:**

Despite a considerable benefit of adding immune checkpoint inhibitors (ICIs) to platinum-based chemotherapy for patients with extensive-stage SCLC (ES-SCLC), a durable response to ICIs occurs in only a small minority of such patients.

**Methods:**

A total of 135 patients with ES-SCLC treated with chemotherapy either alone (chemo-cohort, n = 71) or together with an ICI (ICI combo-cohort, n = 64) was included in this retrospective study. Tumors were classified pathologically as inflamed or noninflamed on the basis of programmed death-ligand 1 expression and CD8^+^ tumor-infiltrating lymphocyte density. Immune-related gene expression profiling was performed, and predicted neoantigen load was determined by whole-exome sequencing.

**Results:**

Among patients in the ICI combo-cohort, median progression-free survival was 10.8 and 5.1 months for those with inflamed (n = 7) or noninflamed (n = 56) tumors, respectively (log-rank test *p* = 0.002; hazard ratio of 0.26). Among the 89 patients with immune-related gene expression profiling data available, inflamed tumors had a higher T cell-inflamed GEP score than did noninflamed tumors (−0.18 versus −0.58, *p* < 0.001). The 12-month progression-free survival rate was 16.1% and 0% for patients in the ICI combo-cohort harboring tumors with a high (n = 26) or low (n = 18) frameshift neoantigen load, respectively. A high-frameshift neoantigen load was associated with up-regulation of gene signatures related to antigen presentation and costimulatory signaling. A durable clinical benefit of ICI therapy was observed only in patients with inflamed tumors and a high-frameshift neoantigen load.

**Conclusions:**

Expression of programmed death-ligand 1, CD8^+^ T cell infiltration, and a high-frameshift neoantigen load are associated with clinical benefit of ICI therapy in ES-SCLC.

**Clinical trial registration:**

UMIN000041056

## Introduction

SCLC is an aggressive high-grade neuroendocrine tumor with a low survival rate. It accounts for approximately 15% of lung cancer cases worldwide and is the sixth leading cause of cancer-related death.^1^, ^2^, ^3^ The standard treatment for SCLC remained unchanged for several decades, with no improvement in survival time.^4^ Recently, however, phase 3 trials have revealed a substantial survival advantage for the addition of antibodies to programmed death-ligand 1 (PD-L1) to first-line chemotherapy for extensive-stage SCLC (ES-SCLC),^5^^,^^6^ although the benefit of this new treatment strategy is restricted to a small subset of patients, in part because of a limited understanding of both the disease and the key determinants of a response to immunotherapy.^7^^,^^8^

SCLC is strongly associated with smoking and therefore has a relatively high tumor mutation burden (TMB), suggesting that it might be responsive to immune checkpoint inhibitors (ICIs).^9^, ^10^, ^11^, ^12^ Nevertheless, only approximately 20% of SCLC tumors have a tumor proportion score for PD-L1 of more than or equal to 1%.^13^, ^14^, ^15^, ^16^ A better understanding of the transcriptomic and genomic features of SCLC is therefore needed to inform the development of optimal therapeutic strategies.

We hypothesized that a comprehensive molecular analysis of the tumor immune microenvironment (TME) and genomic underpinnings of tumor antigenicity for SCLC might reveal immunologic determinants of the response or resistance to immunotherapy and thereby support both the identification of patients likely to derive the most benefit from such treatment and the development of new therapeutic approaches. We have therefore now performed an exploratory study to characterize the pathologic, transcriptomic, and genetic immune profiles of SCLC.

## Materials and Methods

### Patients

We reviewed the medical records of all individuals with pathologically confirmed ES-SCLC treated at the study hospitals between January 2015 and January 2021. Patients diagnosed on the basis of cytology only or with insufficient residual tissue specimens were excluded from biomarker analysis. The chemo-cohort comprised patients treated with platinum-based chemotherapy without an ICI, whereas the ICI combo-cohort comprised those treated with such chemotherapy in combination with an ICI. Among individuals who received prior chemoradiotherapy for limited-stage SCLC, those who had been treated with curative intent and experienced a treatment-free interval of at least 6 months after the last chemotherapy, radiotherapy, or chemoradiotherapy cycle and before the diagnosis of ES-SCLC were also included. The study was conducted according to the Declaration of Helsinki and protocols approved by the institutional review board of each participating hospital (Kindai University Hospital, Kishiwada City Hospital, Izumi City General Hospital, Kurashiki Central Hospital, Osaka City University Hospital, National Hospital Organization Osaka Minami Medical Center, Kindai University Nara Hospital, St. Luke's International Hospital). All patients provided written informed consent, where applicable, or such informed consent was waived by institutional review board–approved protocols for aggregate deidentified data analysis.

### Data Collection

Medical records were reviewed, and data regarding clinicopathologic features and treatment history were extracted. The data cutoff date was June 30, 2021. Tumor response was assessed by computed tomography every 6 to 8 weeks according to Response Evaluation Criteria in Solid Tumors, version 1.1.^17^ Progression-free survival (PFS) was measured from treatment initiation to clinical or radiographic progression or death from any cause. Patients without documented clinical or radiographic disease progression were censored on the date of last follow-up.

### Statistical Analysis

Categorical and continuous variables were summarized descriptively as percentage and median values. Differences in continuous variables were assessed with the Wilcoxon ranked sum test and those in categorical variables with Fisher’s exact test. Comparisons among more than two groups were performed with Dunn’s test. Correlations were evaluated with the Spearman correlation test. The Benjamini-Hochberg method was applied to calculate the false discovery rate (FDR) for multiple testing. Differences in PFS curves constructed by the Kaplan-Meier method were assessed with the log-rank test, and the Cox proportional hazard regression model was adopted to determine hazard ratios (HRs). All *p* values are two-sided and confidence intervals (CIs) are at the 95% level, with statistical significance defined as a *p* value of less than 0.05 (with the exception of Dunn’s test, *p* < 0.025). Statistical analysis was performed with Stata IC version 14.2 (StataCorp LP) or GraphPad Prism 7.0 (GraphPad Software).

### Assessment of Pathologic, Transcriptomic, and Genetic Immune Profiles

Protocols for immunohistochemistry, assessment of immune-related gene expression, and whole-exome sequencing (WES) are described in the Supplementary Methods.

## Results

### Patient Characteristics

A total of 135 patients who were treated between January 2015 and January 2021 and who had baseline tissue specimens available was enrolled, with 71 patients in the chemo-cohort and 64 patients in the ICI combo-cohort. Patient flow is summarized in Supplementary Figure 1. Demographic characteristics were well balanced between the two cohorts (Table 1).

**Table 1:** Characteristics of the Study Patients

Characteristics	Number of Patients (%)^a^		*p* Value^b^
	Chemo-Cohort (n = 71)	ICI Combo-Cohort (n = 64)	
Median age (range), y^c^	73 (35–84)	72 (34–83)	0.384
Sex			
Male	55 (77.5)	53 (82.8)	0.520
Female	16 (22.5)	11 (17.2)	
ECOG performance status			
0–1	54 (76.1)	55 (85.9)	0.293
2	12 (16.9)	5 (7.8)	
3–4	5 (7.0)	4 (6.3)	
Smoking status^d^			
Current or former	68 (95.8)	63 (98.4)	0.687
Never	2 (2.8)	1 (1.6)	
Unknown	1 (1.4)	0 (0)	
Stage			
Limited	8 (11.3)	4 (6.3)	0.374
Extensive	63 (88.7)	60 (93.8)	
Metastasis at baseline			
CNS	17 (23.9)	20 (31.2)	0.440
Intrathoracic only	9 (12.7)	9 (14.1)	1.00
Extrathoracic	54 (76.1)	52 (81.3)	0.532
Histologic diagnosis			
Small cell	66 (93.0)	61 (95.3)	0.721
Combined	5 (7.0)	3 (4.7)	
Treatment			
Surgery	9 (12.7)	4 (6.3)	0.252
Radiotherapy	4 (5.6)	6 (9.4)	0.517
PD-L1 TPS (22C3)			
≥1%	3 (4.2)	3 (4.7)	1.00
<1%	68 (95.8)	61 (95.3)	
PD-L1 CPS (22C3)			
≥1%	18 (25.4)	9 (14.1)	0.132
<1%	53 (74.6)	55 (85.9)	

CNS, central nervous system; CPS, combined positive score; ECOG, Eastern Cooperative Oncology Group; ICI, immune checkpoint inhibitor; PD-L1, programmed death-ligand 1; TPS, tumor proportion score.

a Percentages may not add up to 100 because of rounding.

b *p* Values were determined with the Wilcoxon ranked sum test or Fisher’s exact test as appropriate.

c At the start of treatment.

d Current smokers, individuals who had smoked a cigarette within the previous year; former smokers, those who had smoked more than or equal to 100 cigarettes but had quit more than 1 year before diagnosis; never smokers, those who had smoked less than 100 cigarettes.

### TME Classification on the Basis of PD-L1 Expression and CD8^+^ TIL Density

Median follow-up time was 32.9 months (range: 0.6–37.8 mo) for the chemo-cohort and 15.9 months (range: 1.8–20.8 mo) for the ICI combo-cohort. Median PFS was 4.8 months (95% CI: 4.2–5.3 mo) and 5.3 months (95% CI: 4.6–5.7 mo) in the chemo-cohort and ICI combo-cohort, respectively. The 12-month PFS rate was 4.4% (95% CI: 1.1%–11.1%) and 11.1% (95% CI: 4.9%–20.2%) in the chemo-cohort and ICI combo-cohort, respectively (Supplementary Fig. 2*A* and *B*).

**Figure 1:**
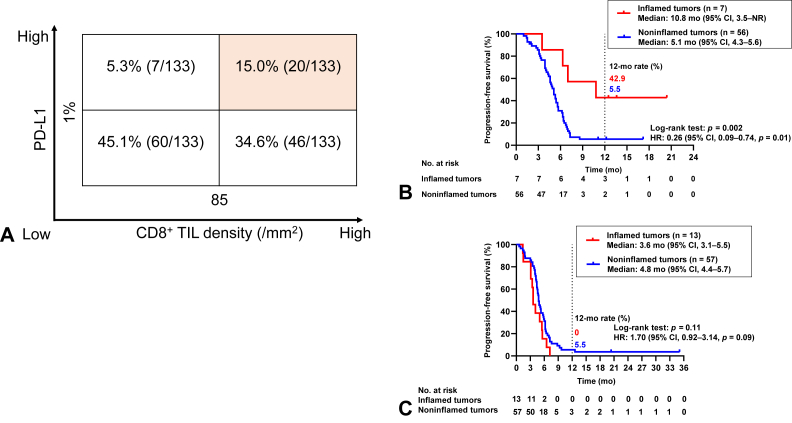
Classification of the tumor immune microenvironment on the basis of PD-L1 expression and CD8^+^ TIL density. (*A*) Tumor immune microenvironment for 133 patients with SCLC classified according to cutoffs for PD-L1 expression (combined positive score) and CD8^+^ TIL density of 1% and the median (85/mm^2^), respectively. Tumors with a PD-L1^positive^ and CD8^+^ TIL^high^ immune microenvironment were designated as inflamed, and all other tumors as noninflamed. (*B*) Kaplan-Meier curves for progression-free survival of patients with inflamed tumors (n = 7) or noninflamed tumors (n = 56) in the ICI combo-cohort. (*C*) Kaplan-Meier curves for progression-free survival of patients with inflamed tumors (n = 13) or noninflamed tumors (n = 57) in the chemo-cohort. CI, confidence interval; HR, hazard ratio; NR, not reached; PD-L1, programmed death-ligand 1; TIL, tumor-infiltrating lymphocyte.

The 133 patients for whom both PD-L1 and CD8 expression data were available were stratified into four TME groups on the basis of cutoffs of 1% for PD-L1 combined positive score (CPS) and of the median (85/mm^2^) for CD8^+^ tumor-infiltrating lymphocyte (TIL) density (Fig. 1*A*). We defined PD-L1^positive^ (CPS of ≥1%) and CD8^+^ TIL^high^ (>85/mm^2^) tumors on the basis of this stratification as “inflamed tumors” and all other tumors as “noninflamed tumors.” For the ICI combo-cohort (n = 63), median PFS was 10.8 months (95% CI: 3.5 mo–not reached; n = 7) in patients with inflamed tumors versus 5.1 months (95% CI: 4.3–5.6 mo; n = 56) in those with noninflamed tumors (log-rank test *p* = 0.002, HR = 0.26, 95% CI: 0.09–0.74), with 12-month PFS rates of 42.9% (95% CI: 9.8%–73.4%) and 5.5% (95% CI: 1.4%–13.7%), respectively (Fig. 1*B*). In contrast, for the chemo-cohort (n = 70), there was no significant difference in PFS between inflamed and noninflamed tumors (median of 3.6 mo [95% CI: 3.1–5.5 mo] versus 4.8 months [95% CI: 4.4–5.7 mo], respectively; log-rank test *p* = 0.11; HR = 1.70, 95% CI: 0.92–3.14), with 12-month PFS rates of 0% and 5.5% (95% CI: 1.5%–13.8%), respectively (Fig. 1*C*). These results suggested that the combination of PD-L1 CPS and CD8^+^ TIL density might serve as a potential biomarker for patient selection with regard to immunotherapy in SCLC.

### Transcriptomic Features of the TME According to PD-L1 Expression and CD8^+^ TIL Density

**Figure 2:**
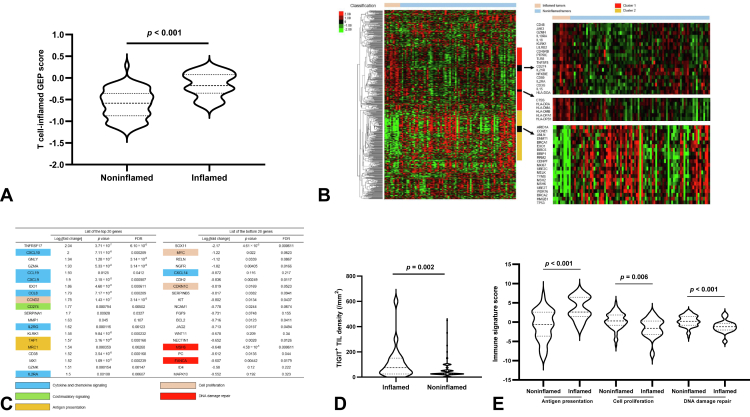
Transcriptomic features of the tumor immune microenvironment. (*A*) Violin plots of the T cell–inflamed GEP score for inflamed tumors (n = 17) and noninflamed tumors (n = 72). The *p* value was determined with the Wilcoxon ranked sum test. (*B*) Heat map of immune-related gene expression in inflamed tumors (n = 17) compared with noninflamed tumors (n = 72). Each colored square represents the Z score for the expression of one gene, with the highest expression illustrated in red, median in black, and lowest in green. Classification of the tumor immune microenvironment as inflamed or noninflamed is found above the heat map, and expanded views for selected genes of interest in clusters 1 and 2 that were preferentially expressed in inflamed and noninflamed tumors, respectively, are found on the right. (*C*) List of the top 20 and bottom 20 genes expressed differentially in inflamed tumors relative to noninflamed tumors as determined from volcano plot analysis (Supplementary Fig. 3*A*). Genes related to antigen presentation, costimulatory signaling, cytokine and chemokine signaling, cell proliferation, or DNA damage repair are shaded as indicated. (*D*) Violin plots of TIGIT^+^ TIL density in inflamed (n = 17) and noninflamed (n = 72) tumors. The *p* value was determined with the Wilcoxon ranked sum test. (*E*) Violin plots for the expression of gene signatures related to antigen presentation, cell proliferation, or DNA damage repair in inflamed (n = 17) and noninflamed (n = 72) tumors. The *p* values were determined with the Wilcoxon ranked sum test. FDR, false discovery rate; GEP, gene expression profiling; TIL, tumor-infiltrating lymphocyte.

We next performed immune-related gene expression profiling (irGEP) for 50 and 39 tumor samples obtained from the chemo-cohort and ICI combo-cohort, respectively, to evaluate the immune profile of SCLC in more detail. A T cell–inflamed GEP score was calculated as a weighted sum of normalized expression values for 18 genes, as described previously,^18^ with this score having been found to be associated with benefit of immunotherapy in solid tumors.^19^ Among the 89 studied patients, the 17 individuals with inflamed tumors had a higher T cell–inflamed GEP score than did the 72 individuals with noninflamed tumors (−0.18 versus −0.58, *p* < 0.001) (Fig. 2*A*).

We further investigated the immunologic characteristics of inflamed tumors (n = 17) and noninflamed tumors (n = 72). We thus performed unsupervised analysis of 676 immune-related genes for the 89 samples subjected to irGEP (Fig. 2*B*). On the basis of the hierarchical clustering for the 89 patients illustrated in Figure 2*B*, we selected two gene clusters that were expressed at a higher level in inflamed tumors (cluster 1, 217 genes) or in noninflamed tumors (cluster 2, 169 genes). Cluster 1 (n = 217 genes) contained genes related to costimulatory T cell signaling (n = 26 genes, including *CD48*, *CD80*, *CD274*, *IL18*, *LILRB2*, *PTPRC*, *IL2RA*, and *IL15*), cytokine and chemokine signaling (n = 25 genes, including *CXCL10*, *IL2RA*, *IL10RA*, and *JAK3*), and antigen presentation (n = 34 genes, including *CTSS* and *HLA-DRA*, *-DMA*, *-DMB*, *-DOA*, *-DPA1*, and *-DPB1*). In contrast, cluster 2 (n = 169 genes) contained genes related to cell proliferation (n = 28 genes, including *ANLN*, *BIRC5*, *CCNE1*, *CENPF*, *MKI67*, *MELK*, *RRM2*, *TYMS*, *TP53*, and *UBE2C*) and DNA damage repair (n = 20 genes, including *BRCA1*, *BRCA2*, *BRIP1*, *EXO1*, *MSH2*, *MSH6*, and *UBE2T*).

We also evaluated differential expression of individual genes with the 89 tumor specimens to shed light on differentially enriched processes in inflamed tumors versus noninflamed tumors. Genes related to cytotoxic lymphocytes (such as *GZMA*), costimulatory molecules (such as *CD274* and *TIGIT*), and cytokine and chemokine signaling (such as *IL2RG*, *IL2RA*, *CXCL9*, and *CXCL10*) were among those expressed at a significantly higher level in inflamed tumors (Fig. 2*C* and Supplementary Fig. 3*A*). In contrast, *SOX11* (*p* < 0.001, FDR < 0.001) and *MYC* (*p* = 0.02, FDR = 0.06) were the top two up-regulated genes in noninflamed tumors relative to inflamed tumors, suggesting *SOX11* and *MYC* might contribute to poor immunoreactivity in SCLC (Fig. 2*C* and Supplementary Fig. 3*A*). We further investigated whether *MYC* might be a determinant of ICI efficacy. Patients in each cohort were divided into two groups according to the median value for *MYC* expression (Supplementary Fig. 4*A* and *B*). For the ICI combo-cohort (n = 39), median PFS was 4.0 months (95% CI: 3.1–5.4 mo; n = 22) in patients with *MYC*^high^ tumors versus 5.3 months (95% CI: 4.6–7.3 mo; n = 17) in those with *MYC*^low^ tumors (log-rank test *p* = 0.028, HR = 2.18, 95% CI: 1.08–4.40), with 12-month PFS rates of 4.6% (95% CI: 0.3%–18.9%) and 23.5% (95% CI: 7.3%–44.9%), respectively. In contrast, for the chemo-cohort (n = 50), there was no significant difference in PFS between *MYC*^high^ and *MYC*^low^ tumors (median of 4.8 mo [95% CI: 3.6–5.5 mo; n = 23] versus 4.9 mo [95% CI: 4.3–5.9 mo; n = 27], respectively; log-rank test *p* = 0.77, HR = 1.09, 95% CI: 0.61–1.94). These results thus indicated that *MYC* expression was negatively associated with ICI efficacy. In addition, patients were divided into two groups according to *MYC*^high^ *SOX11*^high^ and *MYC*^low^ *SOX*11^low^. There was no significant difference in PFS between these two groups in either cohort, likely due in part to the small sample size (data not shown). We also performed an analysis to evaluate any difference in treatment outcome according to the median value of *TIGIT* expression. There was no significant difference in PFS between *TIGIT*^high^ and *TIGIT*^low^ groups of either the ICI combo-cohort or the chemo-cohort (data not shown).

Among the up-regulated genes in inflamed tumors, *TIGIT* encodes a member of the immunoglobulin superfamily of proteins that is expressed on the surface of T cells and natural killer cells and which has recently been evaluated as a potentially targetable immune checkpoint molecule.^20^ We found that the expression level of *TIGIT* was moderately correlated with TIGIT^+^ TIL density as determined by immunohistochemistry (Spearman correlation coefficient [*r*] = 0.32, *p* = 0.006) (Supplementary Fig. 3*B*) and that TIGIT^+^ TIL density was significantly higher in inflamed tumors than in noninflamed tumors (75/mm^2^ [95% CI: 25–144/mm^2^] versus 25/mm^2^ [95% CI: 25–25/mm^2^], *p* = 0.002) (Fig. 2*D* and Supplementary Fig. 3*C*). These findings thus implicated TIGIT as a potentially targetable molecule in inflamed tumors.

Moreover, our data revealed that the expression of gene signatures related to cell proliferation or to DNA damage repair was significantly higher in noninflamed tumors than in inflamed tumors (*p* < 0.001 and *p* < 0.001, respectively) (Fig. 2*E*). The expression of these gene signatures was inversely correlated with that of other immune-related pathway signatures in the 89 tumor specimens analyzed (Supplementary Fig. 3*D*).

Collectively, our irGEP analysis suggested that a T cell–inflamed gene expression profile might play an important role in promoting anticancer immunity, with the increased expression of genes related to costimulatory signaling, cytokine and chemokine signaling, and antigen presentation providing a potential explanation for the more favorable response of inflamed tumors to ICIs. Conversely, up-regulation of gene signatures related to cell proliferation and DNA damage repair might contribute to the acquisition of an immunosuppressive phenotype. The mechanisms by which cell proliferation and DNA damage repair might contribute to ICI efficacy require further investigation.

### Genomic Features of Tumor Antigenicity in SCLC

TMB is an indirect measure of tumor antigenicity and might play a role in the recognition of cancer cells by the immune system.^21^^,^^22^ Tumor neoantigens are mutant peptides generated as a result of genetic mutations and are capable of eliciting an antitumor T cell response.^23^^,^^24^ Although SCLC has a high TMB and would therefore be expected to induce a strong T cell response, the response to ICIs is limited to less than 15% of patients with SCLC.^25^^,^^26^ Neoantigens generated by insertion-deletion (indel) mutations have been found to be enriched relative to those generated by nonsynonymous single-nucleotide variants (nsSNVs) in various cancer types.^21^ Furthermore, a high load of frameshift neoantigens was associated with increased expression of genes related to immune activation, whereas a high load of nsSNV neoantigens was not.^21^ On the basis of these findings, we defined TMB broadly in our study as the total number of SNVs (synonymous and nonsynonymous) and indels per tumor genomic region analyzed. In addition, our bioinformatics pipelines for the prediction of neoantigens focused on those derived from nsSNVs and frameshift indels (fs-indels).

**Figure 3:**
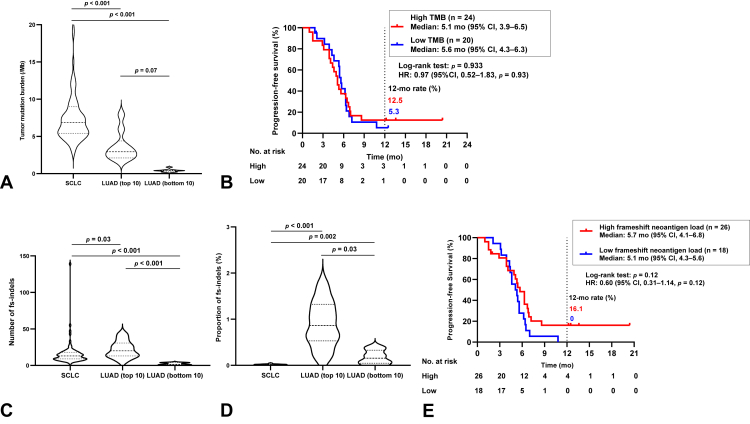
Genomic features of the tumor immune microenvironment. (*A*) Violin plots of tumor mutation burden for SCLC tumors of the present study (n = 85) and for the top 10 and bottom 10 LUAD tumors in TCGA ranked according to TMB. The *p* values were determined with Dunn’s test, with statistical significance defined as a *p* value of less than 0.025. (*B*) Kaplan-Meier curves for progression-free survival according to TMB in the ICI combo-cohort of patients with SCLC. Patients were split into high (≥median) and low (<median) TMB groups. (*C*) Violin plots for the absolute counts of fs-indels in SCLC tumors of the present study (n = 85) and in the top 10 and bottom 10 LUAD tumors in TCGA ranked according to TMB. The *p* values were determined with Dunn’s test, with statistical significance defined as a *p* value of less than 0.025. (*D*) Violin plots for the proportion of fs-indels in SCLC and LUAD as in (*C*). (*E*) Kaplan-Meier curves for progression-free survival according to predicted frameshift neoantigen load in the ICI combo-cohort of patients with SCLC. Patients were split into high (≥median) and low (<median) load groups. CI, confidence interval; fs-indels, frameshift insertions and deletions; HR, hazard ratio; ICI, immune checkpoint inhibitor; LUAD, lung adenocarcinoma; TCGA, The Cancer Genome Atlas; TMB, tumor mutation burden.

We first compared the distribution of TMB between SCLC and lung adenocarcinoma (LUAD), with WES data for 20 patients with LUAD (top 10 and bottom 10 TMB samples) being obtained from The Cancer Genome Atlas (TCGA). As expected, the SCLC tumors in our cohort (n = 85) had a higher TMB compared with LUAD tumors with the top 10 highest TMB values from TCGA (median TMB of 6.8/megabase [Mb] [95% CI: 6.0–7.6/Mb] versus 2.9/Mb [95% CI: 2.1–5.3/Mb], *p* < 0.001) (Fig. 3*A*).

We next evaluated the relation of TMB to PFS in the ICI combo-cohort of our patients with SCLC (n = 44). PFS did not differ significantly between patients with a high versus low TMB (median of 5.1 mo [95% CI: 3.9–6.5 mo] versus 5.6 mo [95% CI: 4.3–6.3 mo], respectively; log-rank test *p* = 0.93; HR = 0.97, with a 95% CI: 0.52–1.83), with 12-month PFS rates of 12.5% (95% CI: 3.1%–28.7%) and 5.3% (95% CI: 0%–21.5%), respectively (Fig. 3*B*). Similarly, in the chemo-cohort (n = 41), the median PFS was 5.3 months (95% CI: 3.8–6.4 mo) for the high-TMB group and 4.4 months (95% CI: 3.2–5.1 mo) for the low-TMB group (log-rank test *p* = 0.18; HR = 0.64, with a 95% CI: 0.34–1.23), with corresponding 12-month PFS rates of 11.8% (95% CI: 0.4%–21.9%) and 0% (Supplementary Fig. 5*A*). These findings suggested that a high TMB was not associated with a clinical benefit of ICI treatment.

We next investigated the potential immunogenicity of nsSNVs and fs-indels in SCLC by prediction of major histocompatibility complex class I–associated neoantigens (Table 2). Analysis of the total of 21,059 nsSNVs detected in the 85 SCLC tumors predicted 3299 high-affinity neoantigens (defined as epitopes with a predicted binding affinity of <50 nM), corresponding to a rate of 0.16 neoantigens per nsSNV. Similar analysis for the total of 1346 fs-indels predicted 662 high-affinity binders, corresponding to a rate of 0.49 neoantigens per fs-indel. Frameshift mutations were thus predicted to generate three times as many neoantigens per mutation as were SNVs, consistent with recent findings for various cancer types.^21^ We performed the same analysis for the TCGA-LUAD data set and found that fs-indels were predicted to give rise to five or 30 times as many neoantigens as were nsSNVs for the top 10 and bottom 10 tumors ranked according to TMB, respectively (Table 2). We then defined the proportion of fs-indels for each tumor as the number of fs-indels divided by the total number of indels and SNVs. The median number of fs-indels tended to be lower in SCLC than in the top 10 LUAD tumors ranked by TMB (13 [95% CI: 10–15] versus 20 [95% CI: 13–34], *p* = 0.03) (Fig. 3*C*), and the proportion of fs-indels in SCLC was significantly lower than in these 10 LUAD tumors (0.018 [95% CI: 0.015–0.021] versus 0.86 [95% CI: 0.41–1.37], *p* < 0.001) (Fig. 3*D*). These findings suggested that the lower number and proportion of fs-indels, and consequent lower load of frameshift neoantigens, might contribute to the limited efficacy of ICIs in SCLC compared with LUAD.

**Table 2:** Predicted Neoantigens for SCLC (This Study) and LUAD (TCGA)

Tumor Type	Mutations (n)	Neoantigens (n)	Neoantigens Per Mutation
SCLC (n = 85)			
nsSNVs	21,059	3299	0.16
fs-indels	1346	662	0.49
fs-indel enrichment			3.14
LUAD (top 10 for TMB)			
nsSNVs	1013	118	0.12
fs-indels	219	132	0.60
fs-indel enrichment			5.17
LUAD (bottom 10 for TMB)			
nsSNVs	38	3	0.08
fs-indels	20	47	2.35
fs-indel enrichment			29.8

LUAD, lung adenocarcinoma; TCGA, The Cancer Genome Atlas; nsSNVs, nonsynonymous single-nucleotide variants; fs-indels, frameshift insertions and deletions; TMB tumor mutation burden.

We next calculated PFS according to predicted frameshift neoantigen load in the ICI combo-cohort of our patients with SCLC (n = 44). The patients were thus split into two groups on the basis of the median number of frameshift neoantigens. The median PFS was 5.7 months (95% CI: 4.1–6.8 mo) for the high-frameshift neoantigen group and 5.1 months (95% CI: 4.3–5.6 mo) for the low-frameshift neoantigen group (log-rank test *p* = 0.12; HR = 0.60, with a 95% CI: 0.31–1.14), with corresponding 12-month PFS rates of 16.1% (95% CI: 5.1%–32.7%) and 0%, respectively (Fig. 3*E*). PFS thus tended to be more favorable for the high-frameshift neoantigen group. In the chemo-cohort (n = 41), median PFS was 5.1 months (95% CI: 4.2–6.0 mo) for the high-frameshift neoantigen group and 4.6 months (95% CI: 3.1-5.6 mo) for the low-frameshift neoantigen group (log-rank test *p* = 0.92, HR = 0.97, 95% CI: 0.51–1.83), with 12-month PFS rates of 5.4% (95% CI: 0.4%–21.9%) and 5.0% (95% CI: 0.4%–20.5%), respectively (Supplementary Fig. 5*B*). Our analysis thus suggested that the number of frameshift neoantigens was more associated with clinical benefit from ICIs than was TMB.

### Association of Immune Signatures Related to Antigen Presentation or Costimulatory Signaling With Frameshift Neoantigens

**Figure 4:**
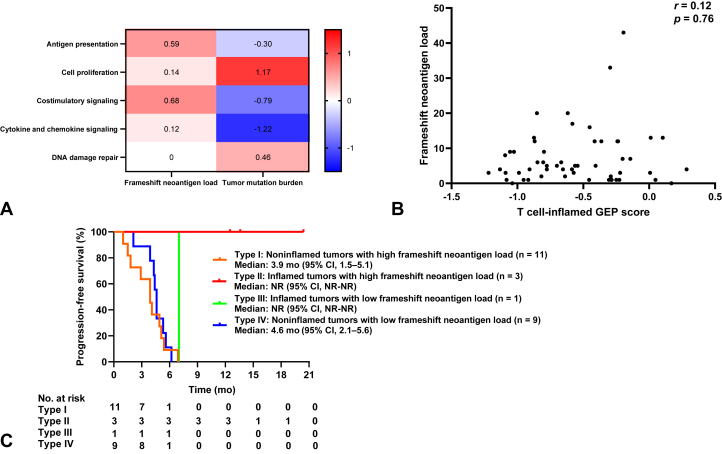
Classification of SCLC tumors on the basis of PD-L1 expression, CD8^+^ tumor-infiltrating lymphocyte density, immune-related gene expression, and neoantigen status. (*A*) Change of median gene signature expression in high versus low groups of SCLC tumors (n = 55) classified according to the median values for the predicted number of frameshift neoantigens or tumor mutation burden. The change of median gene signature expression is represented by the color scale, with the highest values illustrated in red, median in white, and lowest in blue. (*B*) Relation of the predicted number of frameshift neoantigens to the T cell–inflamed GEP score for SCLC tumors (n = 55). The *p* value was determined with the Spearman correlation test. (*C*) Kaplan-Meier curves of progression-free survival for the ICI combo-cohort of patients with SCLC classified according to inflamed versus noninflamed status and predicted frameshift neoantigen load (high defined as ≥5 frameshift neoantigens per case). CI, confidence interval; GEP, gene expression profiling; ICI, immune checkpoint inhibitor; NR, not reached.

To explore further the different effects of TMB and frameshift neoantigen load on immune responses, we evaluated the relation between these two parameters and immune-related gene expression. Patients with SCLC were split into groups on the basis of the median values of TMB (high defined as ≥6.85/Mb) and frameshift neoantigen load (high defined as ≥5 frameshift neoantigens per case). A high load of frameshift neoantigens was associated with a high expression level for immune signatures related to antigen presentation and to costimulatory signaling, whereas a high TMB was associated with a high expression level for immune signatures related to cell proliferation and DNA damage repair (Fig. 4*A*). These findings were similar to the differences in gene expression signatures between inflamed and noninflamed tumors (Fig. 2*B*–*E*), consistent with the notion that frameshift neoantigen load is associated with ICI efficacy in SCLC.

We next evaluated the relation between frameshift neoantigen load and the T cell–inflamed GEP score, which we found was higher in inflamed tumors than in noninflamed tumors (Fig. 2*A*). There was no correlation between these measures (Fig. 4*B*), suggesting the effect of frameshift neoantigen load on ICI efficacy was independent of the T cell–inflamed GEP score.

Finally, we evaluated the clinical utility of the combination of inflammation category (inflamed or noninflamed) and frameshift neoantigen load. Among the 64 patients in the ICI combo-cohort, the 24 individuals with available data were stratified into four groups on the basis of inflammation category and the median frameshift neoantigen load (high defined as ≥5 frameshift neoantigens per case). A durable clinical benefit (PFS of ≥12 mo) was apparent only in patients with both inflamed tumors and a high-frameshift neoantigen load (Fig. 4*C*). Collectively, these results suggested that the expression of PD-L1 and CD8^+^ T cell infiltration, together with a high-frameshift neoantigen load associated with the up-regulation of gene expression signatures related to antigen presentation and costimulatory signaling, might confer a durable clinical benefit of ICI therapy in SCLC.

## Discussion

Our results have revealed that PD-L1 expression and CD8^+^ TIL density together are able to predict which patients with ES-SCLC are likely to derive clinical benefit from the combination of platinum-based chemotherapy and ICI therapy. In addition, a high-frameshift neoantigen load tended to be more associated with clinical benefit from ICI treatment than was a high TMB. Our irGEP analysis implicated a T cell–inflamed TME primed for a response to immunotherapy and up-regulation of gene expression related to costimulatory signaling, cytokine and chemokine signaling, and antigen presentation as determinants of the antitumor immune response.

Although classification of tumors into groups on the basis of PD-L1 status and the presence of TILs has been proposed for other cancer types,^27^^,^^28^ SCLC has not previously been evaluated for the relation between immune characteristics and clinical outcome of chemo-immunotherapy. Not unexpectedly, we found that pathologically inflamed SCLC tumors as defined on the basis of PD-L1 expression level and CD8^+^ TIL density received a greater benefit from anticancer immunotherapy. We also identified additional distinct transcriptomic features of these tumors including the up-regulation of gene expression related to costimulatory signaling, cytokine and chemokine signaling, and antigen presentation.

Consistent with the finding that 12.6% of patients with ES-SCLC received a durable clinical benefit (PFS of ≥12 mo) from chemotherapy plus an ICI in the IMpower133 trial,^5^ 11.1% of the patients with ES-SCLC in our cohort had such a benefit. In our cohort, 85% of patients were classified as having noninflamed tumors. Although a phase 3 trial revealed no benefit from adding an antibody to CTLA-4 and an anti–PD-L1 antibody to platinum-based chemotherapy for patients with ES-SCLC,^8^ our results suggest that the approximately 85% of patients with noninflamed tumors might be amenable to combination therapy designed to promote T cell infiltration. Nevertheless, of our patients with ES-SCLC, 15% were classified as having inflamed tumors, but, among the seven of these patients in the ICI combo-cohort, only three had a durable clinical benefit (PFS of ≥12 mo). Moreover, given that TIGIT^+^ TIL density was significantly higher in inflamed tumors than in noninflamed tumors (*p* = 0.002), the combination of an agent that targets TIGIT with a currently available ICI might be a promising treatment approach. A large phase 3 trial (NCT04256421, SKYSCRAPER-02) of the TIGIT inhibitor tiragolumab in combination with atezolizumab-carboplatin-etoposide for ES-SCLC is currently ongoing.^29^

Our exploratory irGEP analysis provided insight into the mechanisms underlying the limited efficacy of ICIs for SCLC, with gene expression signatures related to DNA damage repair and cell proliferation and the expression of *SOX11* and *MYC* being implicated in the lack of immunogenicity. Previous studies have found that genes related to DNA damage repair are expressed at a higher level in SCLC than in LUAD and that SCLC becomes dependent on such repair pathways for tumor maintenance.^30^, ^31^, ^32^ SOX11 is a neuronal differentiation factor and promotes neuroendocrine differentiation of cancer.^33^^,^^34^ Neuroendocrine-high SCLC was recently found to be associated with reduced levels of immune cell infiltration and expression of immune checkpoint–related molecules including PVR, IDO, major histocompatibility complex class II, and TIM3 compared with neuroendocrine-low SCLC.^35^^,^^36^ SOX11 might therefore contribute to immunosuppression by inducing neuroendocrine differentiation in SCLC. The *MYC* proto-oncogene encodes a transcription factor that is overexpressed in many human cancer types, and dysregulation of MYC signaling is implicated in the molecular and histologic heterogeneity of SCLC.^37^^,^^38^ Although MYC activation may influence the antitumor immune response through regulation of CD47 and PD-L1,^37^ its role in the responsiveness of SCLC to ICI therapy remains under investigation.^39^

TMB has emerged as a potential biomarker for the efficacy of programmed cell death protein 1 inhibitors in several tumor types.^10^ Nevertheless, TMB was not predictive of improvement in overall survival by chemo-immunotherapy in ES-SCLC,^40^ and we found that a high TMB was not associated with clinical benefit from ICI treatment in our cohort. Instead, we found that PFS in the ICI combo-cohort tended to be more favorable for patients whose tumors had a high-frameshift neoantigen load, although the number of patients in this analysis was relatively small. We also found that a high-frameshift neoantigen load was associated with up-regulation of gene expression related to antigen presentation and costimulatory signaling, whereas a high TMB was associated with that of gene expression related to cell proliferation and DNA damage repair. As far as we are aware, our study is the first to have analyzed neoantigen load in SCLC and to suggest the importance of frameshift neoantigens instead of TMB as a determinant of ICI efficacy. The benefit of ICIs for SCLC is limited compared with that apparent for other solid tumor types, despite the high TMB of SCLC attributable to its association with tobacco exposure.^1^ Our neoantigen prediction analysis now suggests that the low number and proportion of fs-indels in SCLC compared with LUAD might account for the limited efficacy of ICIs.

Although a strength of our study is the inclusion of two different treatment cohorts, the chemo-cohort and the ICI combo-cohort, our study also has several limitations. First, the study was retrospective in nature and the number of patients was relatively small, precluding multivariate analysis and analysis of a validation cohort. Nevertheless, with the exception of histologic diagnosis in the ICI combo-cohort and central nervous system metastasis in the chemo-cohort, patient characteristics—including performance status, age, and circulating albumin and lactate dehydrogenase levels—were well balanced between individuals with inflamed or noninflamed tumors in the ICI-combo cohort (Supplementary Table 1) and the chemo-cohort (Supplementary Table 2). Second, our molecular data were derived from conventional bulk analysis, involving the processing of a mixture of cell types, and the study was thus not able to assess tumor heterogeneity. Third, we could not evaluate the relation between clinical outcome and the four SCLC subtypes defined by differential expression of the transcription factors ASCL1, NEUROD1, and POU2F3 or low expression of all three transcription factors (SCLC-A, -N, -P, and -I, respectively).^41^^,^^42^ Retrospective analysis of the IMpower133 trial found that the SCLC-inflamed (SCLC-I) subtype responded best to ICI therapy.^42^ SCLC-I tumors manifest high PD-L1 expression and inflammatory features including high expression of *HLA* genes and genes related to interferon activation and immune checkpoints,^42^ and they are therefore similar to the inflamed tumors in our study. We found that 15% of patients with SCLC had inflamed tumors, similar to the frequency of 18% for SCLC-I tumors in IMpower133.

In conclusion, the classification of ES-SCLC tumors into inflamed and noninflamed subtypes on the basis of PD-L1 expression and CD8^+^ TIL density is a simple approach supported by gene expression analysis to the identification of patients likely to benefit most from the addition of an ICI to chemotherapy. In addition to expression of PD-L1 and CD8^+^ T cell infiltration, a high-frameshift neoantigen load was associated with a durable clinical benefit from ICI therapy in ES-SCLC. Our study thus provides insight into the pathologic, transcriptomic, and genetic immune profiles of SCLC. Further investigation of inflamed and noninflamed tumors should inform personalized treatment strategies and identify treatment resistance mechanisms in SCLC.

## CRediT Authorship Contribution Statement

**Hiroaki Kanemura:** Conceptualization, data curation, formal analysis, funding acquisition, investigation, writing-original draft, writing-review and editing.

**Hidetoshi Hayashi:** Conceptualization, funding acquisition, supervision, investigation, project administration, writing-original draft, writing-review and editing.

**Shuta Tomida:** Formal analysis, investigation, methodology, software, writing-review and editing.

**Junko Tanizaki, Yusuke Kawanaka, Asuka Tsuya, Yasushi Fukuda, Hiroyasu Kaneda, Keita Kudo, Takayuki Takahama, Ryosuke Imai:** Data curation, resources.

**Shinichiro Suzuki:** Data curation, methodology, resources.

**Koji Haratani, Akihiko Ito, Kazuko Sakai, Kazuto Nishio:** Methodology.

**Yasutaka Chiba:** Formal analysis.

**Tomoyuki Otani:** Methodology, investigation.

**Kazuhiko Nakagawa:** Conceptualization, funding acquisition, project administration, supervision, writing-review and editing.
