# Supplementary material for: The Tumor Immune Microenvironment and Frameshift Neoantigen Load Determine Response to PD-L1 Blockade in Extensive-Stage SCLC
Source: JTO Clin Res Rep. 2022 Jul 1;3(8):100373. doi: 10.1016/j.jtocrr.2022.100373 (PMC9356091; doi:10.1016/j.jtocrr.2022.100373)
Supplement: Supplementary Figure S1 [file mmc1.pptx]

## Slide 1
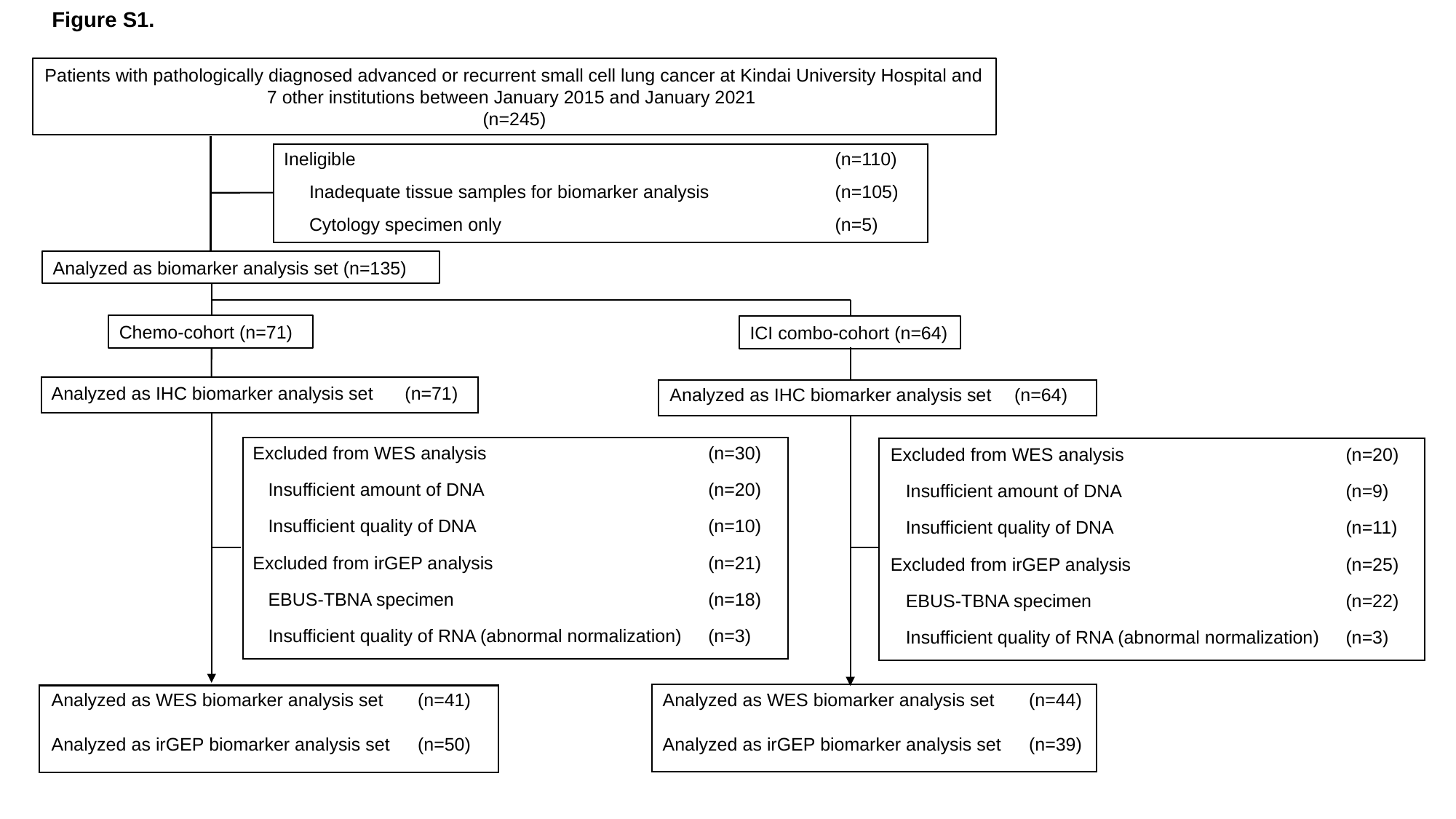

Figure S1.
Patients with pathologically diagnosed advanced or recurrent small cell lung cancer at Kindai University Hospital and
7 other institutions between January 2015 and January 2021
(n=245)
| Ineligible | (n=110) |
| --- | --- |
| Inadequate tissue samples for biomarker analysis | (n=105) |
| Cytology specimen only | (n=5) |
Analyzed as biomarker analysis set (n=135)
Chemo-cohort (n=71)
ICI combo-cohort (n=64)
| Analyzed as IHC biomarker analysis set | (n=71) |
| --- | --- |
| Analyzed as IHC biomarker analysis set | (n=64) |
| --- | --- |
| Excluded from WES analysis | (n=30) |
| --- | --- |
| Insufficient amount of DNA | (n=20) |
| Insufficient quality of DNA | (n=10) |
| Excluded from irGEP analysis | (n=21) |
| EBUS-TBNA specimen | (n=18) |
| Insufficient quality of RNA (abnormal normalization) | (n=3) |
| Excluded from WES analysis | (n=20) |
| --- | --- |
| Insufficient amount of DNA | (n=9) |
| Insufficient quality of DNA | (n=11) |
| Excluded from irGEP analysis | (n=25) |
| EBUS-TBNA specimen | (n=22) |
| Insufficient quality of RNA (abnormal normalization) | (n=3) |
| Analyzed as WES biomarker analysis set | (n=41) |
| --- | --- |
| Analyzed as irGEP biomarker analysis set | (n=50) |
| Analyzed as WES biomarker analysis set | (n=44) |
| --- | --- |
| Analyzed as irGEP biomarker analysis set | (n=39) |
