# Supplementary figures and images for: The Tumor Immune Microenvironment and Frameshift Neoantigen Load Determine Response to PD-L1 Blockade in Extensive-Stage SCLC
Source: JTO Clin Res Rep. 2022 Jul 1;3(8):100373. doi: 10.1016/j.jtocrr.2022.100373 (PMC9356091; doi:10.1016/j.jtocrr.2022.100373)

## Slide 1
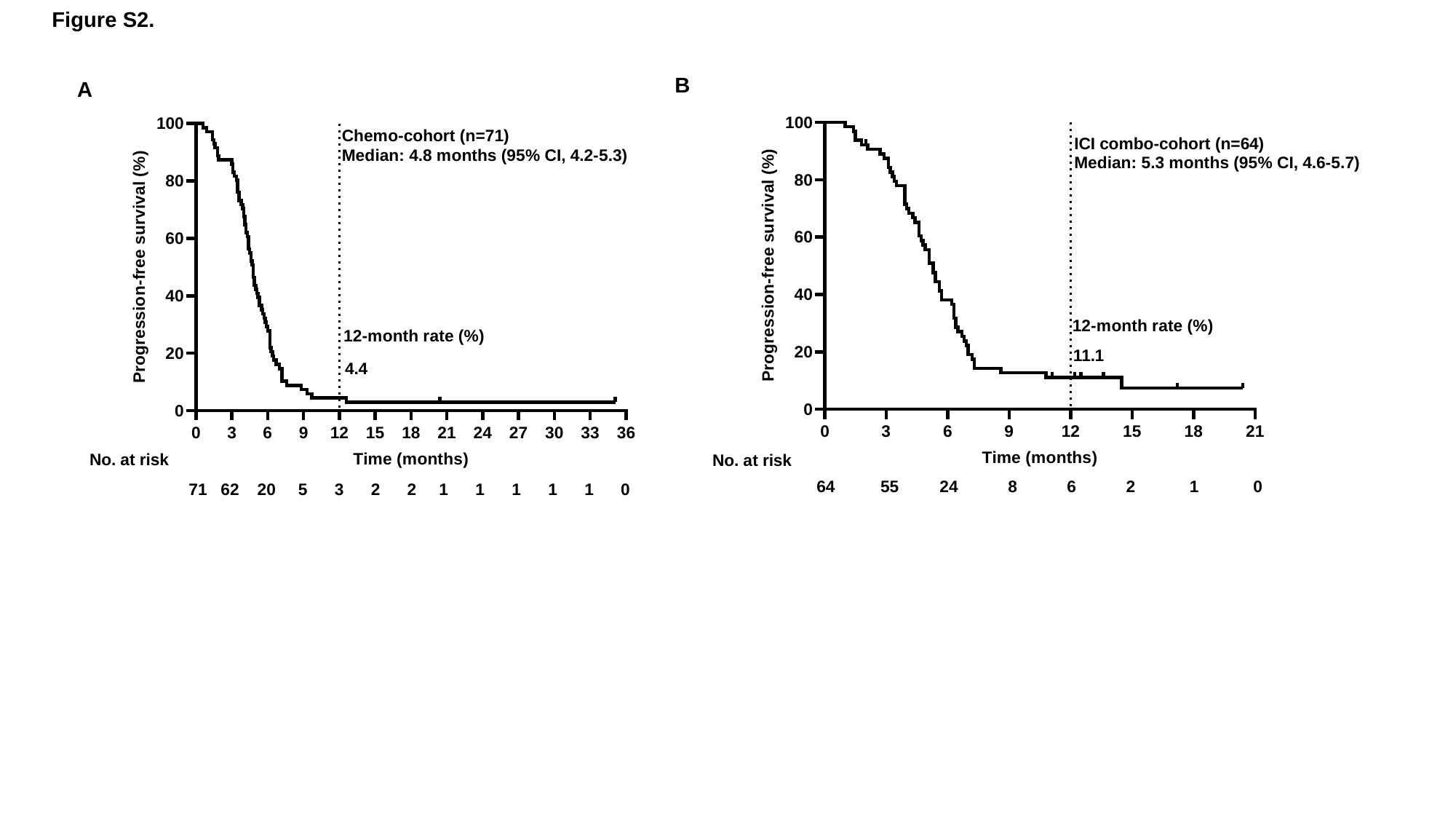

Figure S2.
B
A

Supplement: Supplementary Figure S2 [file mmc2.pptx]

## Slide 1
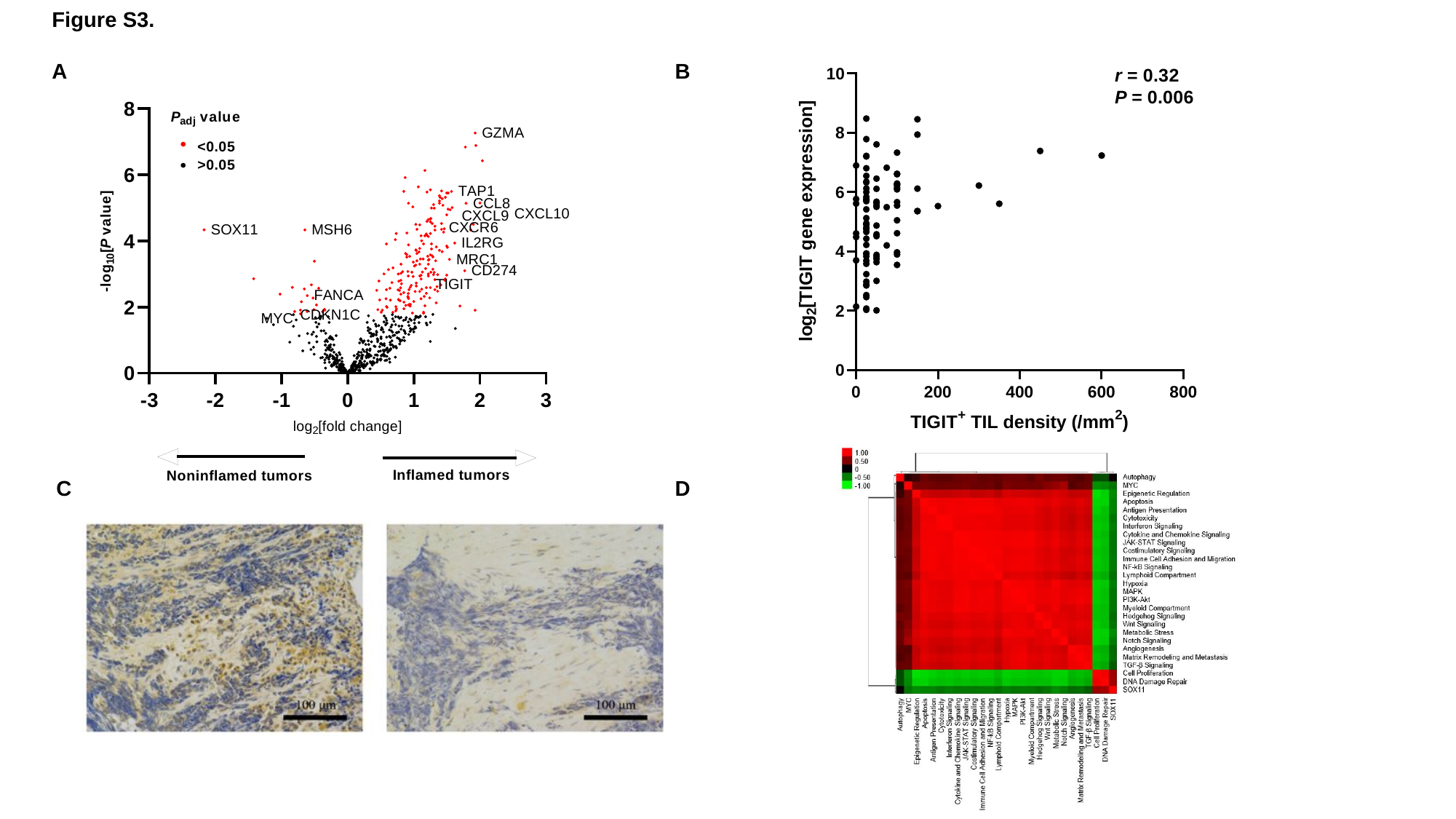

Figure S3.
A
B
C
D

Supplement: Supplementary Figure S3 [file mmc3.pptx]

## Slide 1
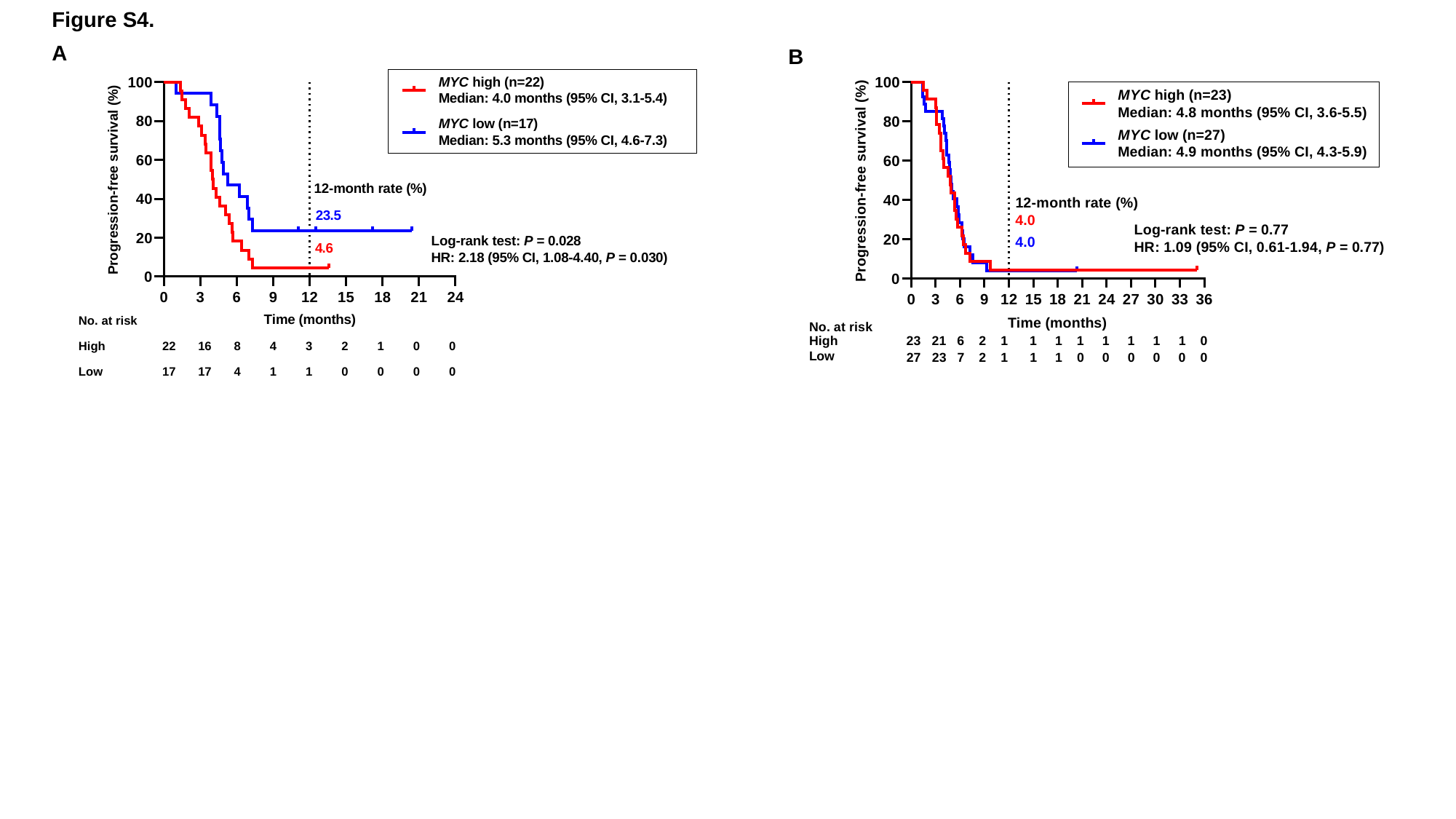

Figure S4.
A
B
| No. at risk | | | | | | | | | |
| --- | --- | --- | --- | --- | --- | --- | --- | --- | --- |
| High | 22 | 16 | 8 | 4 | 3 | 2 | 1 | 0 | 0 |
| Low | 17 | 17 | 4 | 1 | 1 | 0 | 0 | 0 | 0 |

Supplement: Supplementary Figure S4 [file mmc4.pptx]

## Slide 1
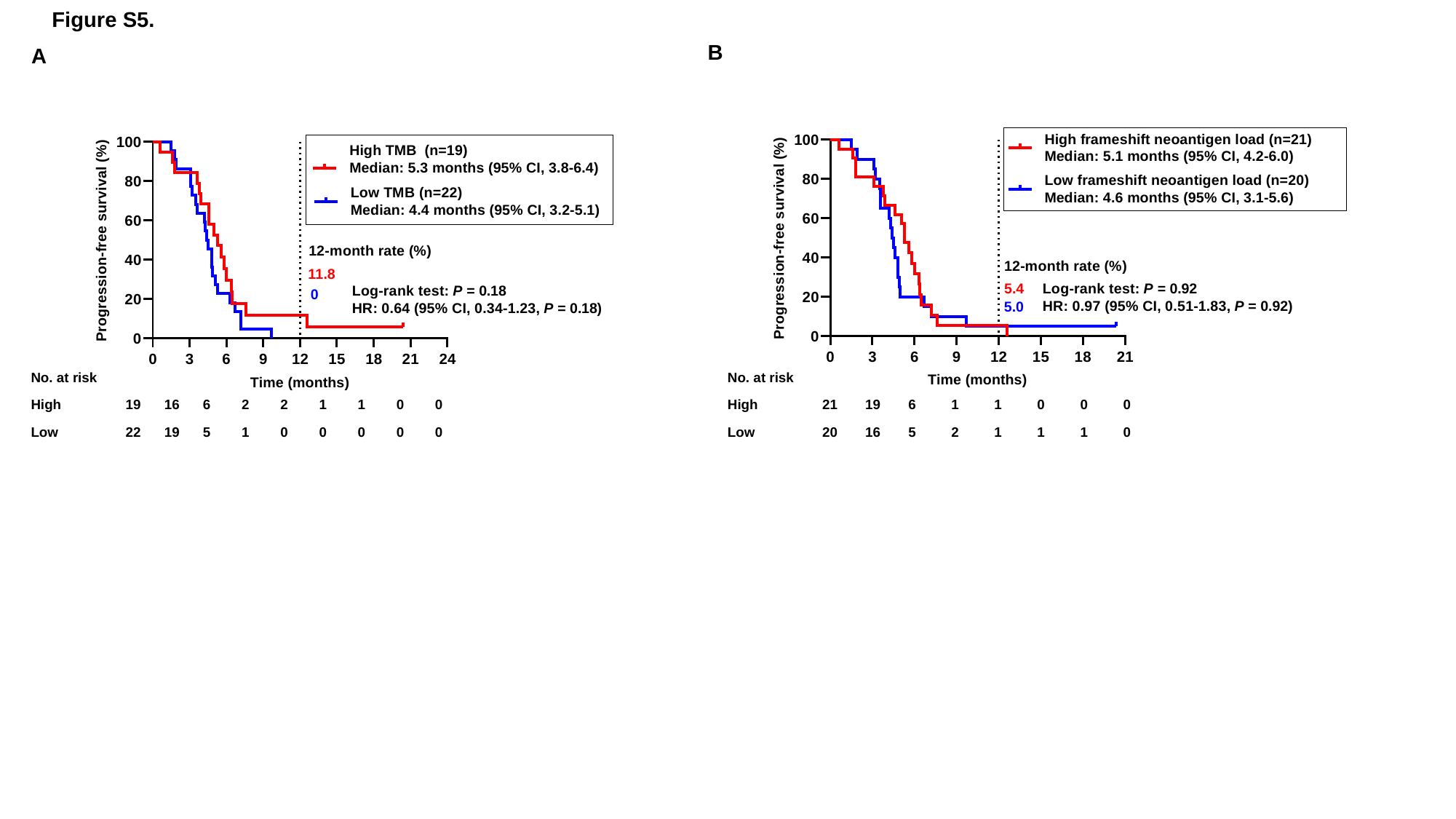

Figure S5.
B
A
| No. at risk | | | | | | | | | |
| --- | --- | --- | --- | --- | --- | --- | --- | --- | --- |
| High | 19 | 16 | 6 | 2 | 2 | 1 | 1 | 0 | 0 |
| Low | 22 | 19 | 5 | 1 | 0 | 0 | 0 | 0 | 0 |
| No. at risk | | | | | | | | |
| --- | --- | --- | --- | --- | --- | --- | --- | --- |
| High | 21 | 19 | 6 | 1 | 1 | 0 | 0 | 0 |
| Low | 20 | 16 | 5 | 2 | 1 | 1 | 1 | 0 |

Supplement: Supplementary Figure S5 [file mmc5.pptx]
