# Supplementary material for: The Tumor Immune Microenvironment and Frameshift Neoantigen Load Determine Response to PD-L1 Blockade in Extensive-Stage SCLC
Source: JTO Clin Res Rep. 2022 Jul 1;3(8):100373. doi: 10.1016/j.jtocrr.2022.100373 (PMC9356091; doi:10.1016/j.jtocrr.2022.100373)
Supplement: Supplementary Methods [file mmc6.docx]

**Supplementary Methods**

**Immunohistochemistry**

Tumor histology was classified according to WHO criteria.^1^ Sections of formalin-fixed paraffin-embedded (FFPE) tumor tissue (thickness, 4 μm) from patients in the IHC biomarker analysis set were subjected to immunohistochemistry (IHC) with monoclonal antibodies to PD-L1 (kit with clone 22C3, Agilent Technologies), to CD8 (clone C8/144B, Agilent Technologies), and to TIGIT (clone TG1, Oncodianova) and with the use of an automated stainer (Autostainer Link 48 and Leica Bond-Max). The stained slides were evaluated by a board-certified pathologist who was blinded to clinical outcome.

PD-L1 immunostaining was optimized with human placenta and tonsil as positive controls. The percentage of tumor cells positive for PD-L1 was determined as the PD-L1 tumor proportion score (TPS).^2, 3^ The combined positive score (CPS) for PD-L1 expression was also calculated as the number of PD-L1–positive cells (tumor cells, lymphocytes, macrophages) divided by the total number of tumor cells and multiplied by 100.^4, 5^ PD-L1 positivity was defined as membranous staining at any intensity with a cutoff of ≥1% of tumor cells (<1% defined as negative).

Tumor-infiltrating lymphocytes (TILs) were evaluated on the basis of staining for CD8 and TIGIT. Tumor tissue samples including at least 100 viable tumor cells were eligible for TIL assessment. The number of TILs was determined at an absolute magnification of 400× (0.20 mm^2^ per field). At least one and a maximum of five scanned fields of tumor regions were randomly chosen for each TIL count. TILs were counted by a board-certified pathologist, and the density of TILs in each tumor was calculated by dividing the number of TILs by the sum of the area (mm^2^) of the viewed fields.^3^ TILs were defined as cells positive for CD8 or TIGIT at any staining intensity.

**Immune-related gene expression profiling**

Immune-related gene expression profiling (irGEP) was performed with RNA isolated from baseline FFPE tumor samples. Sections were first examined by hematoxylin-eosin staining to confirm the presence of invasive tumor cells and to determine the tumor area. Macrodissection was performed when needed to avoid contamination with normal tissue. Tissue obtained by transbronchial needle aspiration and cell block specimens were excluded to avoid contamination by non–tumor-infiltrating immune cells. Total RNA was extracted from the dissected FFPE tumor tissue with the use of an AllPrep DNA/RNA FFPE Kit (Qiagen). The amount of extracted RNA was measured with a NanoDrop 2000 device (Thermo Fisher Scientific) and Ribogreen RNA Assay Kit (Thermo Fisher Scientific). The integrity of the RNA was assessed with an Agilent RNA 6000 Nano Kit and an Agilent 2100 Bioanalyzer (Agilent Technologies), and the percentage of fragments comprising ≥300 nucleotides (DV300) was calculated. A minimum of 50 ng of total RNA was used for gene expression analysis with the nCounter platform and a PanCancer IO 360 Gene Expression Panel comprising 750 immune-related genes and 20 housekeeping genes (NanoString Technologies). Tumor-derived RNA obtained from 95 patients was thus analyzed. Gene expression was normalized on the basis of the data for the 20 housekeeping genes with the use of nSolver Analysis Software 4.0 and nCounter Advanced Analysis 2.0 (NanoString Technologies). Samples with abnormal normalized expression values (normalization factor of >10 obtained with nSolver Analysis Software 4.0) were excluded, in accordance with the manufacturer’s instructions. A total of 89 RNA samples thus remained for further analysis. Of the 750 immune-related genes analyzed, 74 genes for which >60% of samples showed an expression value below the minimum threshold were filtered out. The normalized gene expression data were log_2_-transformed before calculation of the Z score. Gene clustering was performed with the use of Cluster3.0 software, and a heat map was constructed with the use of Java TreeView.^6^

The T cell–inflamed GEP score was based on the expression of 18 inflammatory genes related to antigen presentation, chemokine expression, cytolytic activity, and adaptive immune resistance: *CCL5, CD27, CD274 (PD-L1), CD276 (B7-H3), CD8A, CMKLR1, CXCL9, CXCR6, HLA-DQA1, HLA-DQB1, HLA-E, IDO1, LAG3, NKG7, PDCD1LG2 (PD-L2), PSMB10, STAT1,* *TIGIT*. The score was calculated as the weighted sum of the normalized expression values for these 18 genes.^7, 8^

Immune signatures representative of 11 immune cell types and 25 immune-related pathways were curated as in previous studies.^9-11^ The cell type signatures and corresponding genes were as follows. Cytotoxic cells (7 genes): *NKG7, KLRK1, PRF1, GZMA, KLRD1, KLRB1, GNLY*. Macrophages (4 genes): *CD68, CD84, MS4A4A, CD163*. Dendritic cells (2 genes): *CD209, HSD11B1*. Exhausted CD8 cells (3 genes): *LAG3, EOMES, PTGER4*. B cells (5 genes): *TNFRSF17, MS4A1, CD19, SPIB, FAM30A*. CD45 cells (3 genes): *CD45RA, PTPRC, CD45RO*. Neutrophils (4 genes): *FPR1, FCGR3A/B, CSF3R, CEACAM3*. T cells (5 genes): *TRAT1, CD3D, CD6, CD3E, SH2D1A*. Mast cells (2 genes): *TPSAB1/B2, CPA3*. CD8 T cells (2 genes): *CD8A, CD8B*. Natural killer cells (1 gene): *XCL1/2*. The pathway signatures and corresponding genes were as follows: Angiogenesis (35 genes): *ANGPT1, ANGPT2, ANGPTL4, CCND2, CCNE1, CES3, DLL4, E2F3, EDN1, EZH2, FGF18, FGFR1, FLT1, FSTL3, HEY1, ITGAV, ITPK1, JAG1, MMP9, MMRN2, NFIL3, PDGFB, PGPEP1, RPL7A, SERPINB5, SERPINH1, STC1, THBS1, TNFAIP6, TPM1, TYMP, VCAN, VEGFA, VEGFB, VEGFC*. Antigen presentation (51 genes): *ATF3, B2M, BATF3, CCL4, CCR5, CD36, CD4, CD74, CD8A, CD8B, CDC20, CTSS, CXCL1, CYBB, DTX3L, FCGR1A, HLA-A, HLA-B, HLA-C, HLA-DMA, HLA-DMB, HLA-DOA, HLA-DOB, HLA-DPA1, HLA-DPB1, HLA-DQA1, HLA-DQB1, HLA-DRA, HLA-DRB1, HLA-E, HLA-F, IRF8, ITGAV, KIF2C, KLRD1, MRC1, PSMB10, PSMB5, PSMB8, PSMB9, SOCS1, TAP1, TAP2, TAPBP, THBD, TNF, TRIM21, UBA7, UBE2C, ULBP2, VHL*. Apoptosis (34 genes): *AKT1, APC, BAD, BAX, BBC3, BCL2, BCL2L1, BCL6B, BID, BIRC5, BLM, CASP1, CASP3, CASP8, CASP9, CD14, CDH1, CTNNB1, FADD, HMGB1, LY96, PSMB10, PSMB5, PSMB8, PSMB9, RIPK1, RIPK3, ROCK1, TICAM1, TLR3, TLR4, TNFRSF10B, TNFSF10, TP53*. Autophagy (23 genes): *AKT1, BAD, BCL2, BCL2L1, BNIP3, DEPTOR, HIF1A, HMGB1, HRAS, KRAS, MAP3K7, MAPK10, MTOR, NRAS, PIK3CA, PIK3CD, PIK3R1, PIK3R2, PRKAA2, PRKACB, PTEN, RPS6KB1, RPTOR*. Cell proliferation (48 genes): *ANLN, ATM, BIRC5, BLM, BRCA1, BRCA2, CCNA1, CCNB1, CCND1, CCND2, CCND3, CCNE1, CCNO, CDC20, CDC25C, CDK2, CDK6, CDKN1A, CDKN1C, CDKN2A, CDKN2B, CENPF, CEP55, E2F3, EXO1, H2AFX, KIF2C, MELK, MKI67, MLH1, MYC, NBN, PIAS4, POLD1, PRKCA, PSMB10, PSMB5, PSMB8, PSMB9, RAD50, RAD51, RAD51C, RB1, RBL2, RRM2, TP53, TYMS, UBE2C*. Costimulatory signaling (73 genes): *ADORA2A, AKT1, CD2, CD247, CD27, CD274, CD28, CD3D, CD3E, CD4, CD40, CD44, CD48, CD69, CD80, CD86, CHUK, CTLA4, DPP4, EGR1, FYN, HAVCR2, HLA-DPA1, HLA-DPB1, HLA-DQA1, HLA-DQB1, HLA-DRA, HLA-DRB1, ICOSLG, IKBKB, IKBKG, IL15, IL18, IL18R1, IL2RA, IL2RB, IL2RG, LAG3, LCK, LILRB2, MAP3K7, MAP3K8, MTOR, NECTIN2, NFATC2, NFKB1, NFKBIA, PDCD1LG2, PIK3CA, PIK3R1, PIK3R2, PRR5, PSMB10, PSMB5, PSMB8, PSMB9, PTEN, PTGS2, PTPN11, PTPRC, PVRIG, RELA, RICTOR, RIPK2, SPP1, STAT4, TIGIT, TNFRSF14, TNFRSF25, TRAT1, TSLP, VTCN1, ZAP70*. Cytokine and chemokine signaling (80 genes): *AKT1, CCL14, CCL18, CCL19, CCL2, CCL20, CCL21, CCL3/L1, CCL4, CCL5, CCL8, CCR2, CCR4, CCR5, CHUK, CSF1, CSF1R, CSF2RB, CSF3R, CX3CL1, CX3CR1, CXCL1, CXCL10, CXCL12, CXCL13, CXCL14, CXCL16, CXCL2, CXCL3, CXCL6, CXCL8, CXCL9, CXCR2, CXCR4, CXCR6, GNG4, HCK, HRAS, IKBKB, IKBKG, IL10RA, IL11, IL11RA, IL12RB2, IL15, IL16, IL18, IL18R1, IL1A, IL1B, IL1R2, IL22RA1, IL2RA, IL2RB, IL2RG, IL32, IL33, IL34, IL6, IL6R, IL7R, JAK2, JAK3, KRAS, NFKB1, NFKBIA, NRAS, PIK3CA, PIK3CD, PIK3CG, PIK3R1, PIK3R2, PIK3R5, PRKACB, RELA, ROCK1, SHC2, STAT1, STAT2, STAT3*. Cytotoxicity (42 genes): *BBC3, CBLC, CD47, GHR, GNLY, GZMA, GZMK, IFI16, IFI27, IFI35, IFI6, IFIH1, IFIT1, IFIT2, IFIT3, IFITM1, IFITM2, IGF2R, IL11RA, IL12RB2, IL22RA1, IRF1, IRF4, IRF9, ISG15, JAK1, JAK2, JAK3, KLRB1, KLRD1, KLRK1, LIF, MX1, OAS1, OAS2, OAS3, PRF1, SIRPA, SPRY4, STAT1, STAT2, TNFSF10*. DNA damage repair (31 genes): *ATM, BLM, BRCA1, BRCA2, BRIP1, CCNA1, CCNO, CDK2, DDB2, EXO1, FANCA, H2AFX, ISG15, MGMT, MLH1, MSH2, MSH6, NBN, NEIL1, PARP4, PIAS4, PMS2, POLD1, RAD50, RAD51, RAD51C, TNKS, TP53, UBA7, UBE2T, XCL1/2*. Epigenetic regulation (17 genes): *ARID1A, BNIP3, BRD3, BRD4, CCND1, DNMT1, EZH2, H2AFX, HDAC11, HDAC3, HDAC4, HDAC5, HELLS, HMGA1, JAK2, KAT2B, MAP3K12*. Hedgehog signaling (14 genes): *BMP2, GAS1, GLI1, PRKACB, PSMB10, PSMB5, PSMB8, PSMB9, WNT10A, WNT11, WNT2B, WNT5A, WNT5B, WNT7B*. Hypoxia (39 genes): *AKT1, ALDOA, ANGPT1, ANGPT2, BCL2, CDKN1A, CYBB, EDN1, EGFR, EIF4EBP1, ENO1, ERBB2, FLT1, HIF1A, HK1, HK2, IFNGR1, IFNGR2, IL6, IL6R, LDHA, MTOR, NFKB1, PDK1, PFKFB3, PIK3CA, PIK3CD, PIK3CG, PIK3R1, PIK3R2, PIK3R5, PRKCA, RELA, RPS6KB1, SLC2A1, STAT3, TLR4, VEGFA, VHL*. Immune cell adhesion and migration (80 genes): *CD2, CD274, CD276, CD28, CD4, CD40, CD58, CD6, CD80, CD86, CD8A, CD8B, CDH1, CDH2, CDH5, CLEC14A, CLEC7A, CLECL1, CTLA4, CTNNB1, CXCL12, CXCR4, CYBB, HLA-A, HLA-B, HLA-C, HLA-DMA, HLA-DMB, HLA-DOA, HLA-DOB, HLA-DPA1, HLA-DPB1, HLA-DQA1, HLA-DQB1, HLA-DRA, HLA-DRB1, HLA-E, HLA-F, ICAM1, ICAM2, ICAM3, ICOSLG, ITGA1, ITGA2, ITGA4, ITGA6, ITGAE, ITGAL, ITGAM, ITGAV, ITGAX, ITGB2, ITGB3, ITGB8, MMP9, NCAM1, NECTIN1, NECTIN2, PDCD1LG2, PECAM1, PIK3CA, PIK3CD, PIK3CG, PIK3R1, PIK3R2, PIK3R5, PRKCA, PTPN11, PTPRC, PVR, ROCK1, SELE, SELL, SELP, SIGLEC1, THY1, TIGIT, VCAM1, VCAN, VTCN1*. Interferon signaling (62 genes): *B2M, CD44, EGR1, EIF2AK2, FCGR1A, FLNB, GBP1, GBP2, GBP4, GHR, HLA-A, HLA-B, HLA-C, HLA-DPA1, HLA-DPB1, HLA-DQA1, HLA-DQB1, HLA-DRA, HLA-DRB1, HLA-E, HLA-F, ICAM1, IFI16, IFI27, IFI35, IFI6, IFIH1, IFIT1, IFIT2, IFIT3, IFITM1, IFITM2, IFNAR1, IFNGR1, IFNGR2, IGF2R, IRF1, IRF2, IRF3, IRF4, IRF5, IRF7, IRF8, IRF9, ISG15, JAK1, JAK2, MX1, NCAM1, OAS1, OAS2, OAS3, OASL, PSMB8, PTPN11, RSAD2, SOCS1, STAT1, STAT2, TRIM21, UBA7, VCAM1*. JAK-STAT signaling (47 genes): *AKT1, BCL2, BCL2L1, CCND1, CCND2, CCND3, CDKN1A, CSF2RB, CSF3R, GHR, HRAS, IFNAR1, IFNGR1, IFNGR2, IL10RA, IL11, IL11RA, IL12RB2, IL15, IL22RA1, IL2RA, IL2RB, IL2RG, IL6, IL6R, IL7R, IRF9, JAK1, JAK2, JAK3, LIF, MTOR, MYC, PIAS4, PIK3CA, PIK3CD, PIK3CG, PIK3R1, PIK3R2, PIK3R5, PTPN11, SOCS1, STAT1, STAT2, STAT3, STAT4, TSLP*. Lymphoid compartment (65 genes): *CCR4, CD19, CD2, CD27, CD274, CD28, CD38, CD3D, CD3E, CD40, CD48, CD5, CD6, CD7, CD79A, CD80, CD86, CD8A, CD8B, CD96, CTLA4, CX3CL1, CXCL10, CXCL13, CXCL16, CXCL9, DPP4, EGR1, EOMES, F2RL1, GNLY, GZMA, GZMK, HLA-DOB, ICOSLG, IDO1, IFI27, IFIT1, IFITM1, IGF2R, IL11, IL12RB2, IL18R1, IL2RG, IRF4, IRF9, ISG15, ITGA1, JAK1, JAK2, KLRB1, KLRD1, KLRK1, LAG3, LCK, MS4A1, MX1, PRF1, PVR, SLAMF7, STAT1, STAT2, TIGIT, TNFRSF25, ZAP70*. MAPK (72 genes): *AKT1, ANGPT1, ANGPT2, BAD, BCL2L1, CASP3, CD14, CHUK, CSF1, CSF1R, DUSP1, DUSP2, DUSP5, EGFR, FAS, FGF13, FGF18, FGF9, FGFR1, FLNB, FLT1, GNG4, HRAS, IKBKB, IKBKG, IL1A, IL1B, IL1R2, KDR, KIT, KRAS, MAP3K12, MAP3K5, MAP3K7, MAP3K8, MAPK10, MET, MYC, NF1, NFKB1, NFKB2, NGFR, NRAS, PDGFA, PDGFB, PDGFRB, PIK3CA, PIK3CD, PIK3CG, PIK3R1, PIK3R2, PIK3R5, PLA1A, PRKACB, PRKCA, PTPN11, RASAL1, RELA, RELB, SHC2, TGFB1, TGFB2, TGFB3, TGFBR1, TGFBR2, TNF, TNFRSF1A, TP53, VEGFA, VEGFB, VEGFC, ZAP70*. Matrix remodeling and metastasis (54 genes): *A2M, BMP2, CASP3, CD36, CD44, CD47, CDH1, COL11A1, COL17A1, COL4A5, COL5A1, COL6A3, COMP, CTSS, ICAM1, ICAM2, ICAM3, ITGA1, ITGA2, ITGA4, ITGA6, ITGAE, ITGAL, ITGAM, ITGAV, ITGAX, ITGB2, ITGB3, ITGB8, KDR, LAMA1, LAMB3, LAMC2, LOXL2, LTBP1, MMP1, MMP7, MMP9, NCAM1, NID2, PDGFA, PDGFB, PECAM1, PLOD2, PRKCA, RELN, SERPINH1, SPP1, TGFB1, TGFB2, TGFB3, THBS1, VCAM1, VCAN*. Metabolic stress (82 genes): *AKT1, AQP9, ATM, CCNA1, CCNE1, CD300A, CDK2, CDK6, CDKN1A, CDKN2A, CDKN2B, CEBPB, CXCL8, DEPTOR, E2F3, EGFR, EIF4EBP1, ENO1, ERBB2, ERO1A, EZH2, FBP1, FGFR1, GLS, GOT1, GOT2, H2AFX, HIF1A, HK1, HK2, HMGA1, HRAS, IKBKB, IL1A, IL6, KIT, KRAS, LDHA, LDHB, MAP3K5, MAPK10, MET, MTOR, MYC, NBN, NFKB1, NRAS, PC, PCK2, PDGFRB, PDK1, PFKFB3, PFKM, PIK3CA, PIK3CD, PIK3CG, PIK3R1, PIK3R2, PIK3R5, PKM, PRKAA2, PRKCA, PRR5, PTEN, RAD50, RB1, RELA, RICTOR, RPS6KB1, RPTOR, SGK1, SLC16A1, SLC1A5, SLC2A1, SLC7A5, STAT3, TNF, TP53, TPI1, UBE2C, VEGFA, VHL*. Myeloid compartment (63 genes): *ANGPT1, C5AR1, CCL2, CCL20, CCL4, CCL5, CCL8, CD14, CD47, CDKN1A, CEBPB, CLEC7A, COL11A1, COL17A1, CRABP2, CSF1, CSF1R, CSF3R, CXCL1, CXCL12, CXCL2, CXCL3, CXCL6, CYBB, DAB2, DLL4, FCGR1A, FCN1, FOSL1, FPR1, FPR3, HCK, IER3, IL1A, IL1B, ITGAM, ITGAX, LAMB3, LIF, LILRA5, LILRB2, LY96, LYZ, MMP1, MRC1, NFAM1, NLRP3, P2RY13, PDZK1IP1, PTGS2, S100A8, S100A9, SERPINA1, SIRPA, SIRPB2, SLC11A1, TLR1, TLR2, TLR4, TLR8, TNFAIP6, TREM1, TREM2*. NF-κB signaling (31 genes): *CD27, CD40, CHUK, IKBKB, IKBKG, LTB, NFKB1, NFKB2, NFKBIA, NFKBIE, PSMB10, PSMB5, PSMB8, PSMB9, RELA, RELB, RELN, TNF, TNFRSF11A, TNFRSF11B, TNFRSF14, TNFRSF17, TNFRSF18, TNFRSF1A, TNFRSF1B, TNFRSF25, TNFRSF4, TNFSF12, TNFSF13, TNFSF13B, TNFSF4*. Notch signaling (23 genes): *APH1B, CCND1, DLL1, DLL4, DTX3L, DTX4, E2F3, HDAC11, HDAC3, HDAC4, HDAC5, HES1, HEY1, HIF1A, JAG1, JAG2, KAT2B, MAML2, MFNG, MYC, NOTCH1, NOTCH2, TP53*. PI3K-Akt (92 genes): *AKT1, ANGPT1, ANGPT2, BAD, BCL2, BCL2L1, BRCA1, CASP9, CCND1, CCND2, CCND3, CCNE1, CD19, CDK2, CDK6, CDKN1A, CHUK, COL4A5, COL6A3, COMP, CSF1, CSF1R, CSF3R, EGFR, EIF4EBP1, FGF13, FGF18, FGF9, FGFR1, FLT1, GHR, GNG4, HRAS, IFNAR1, IKBKB, IKBKG, IL2RA, IL2RB, IL2RG, IL6, IL6R, IL7R, ITGA1, ITGA2, ITGA4, ITGA6, ITGAV, ITGB3, ITGB8, JAK1, JAK2, JAK3, KDR, KIT, KRAS, LAMA1, LAMB3, LAMC2, MET, MTOR, MYC, NFKB1, NGFR, NRAS, PCK2, PDGFA, PDGFB, PDGFRB, PIK3CA, PIK3CD, PIK3CG, PIK3R1, PIK3R2, PIK3R5, PRKAA2, PRKCA, PTEN, RBL2, RELA, RELN, RPS6KB1, RPTOR, SGK1, SPP1, SYK, THBS1, TLR2, TLR4, TP53, VEGFA, VEGFB, VEGFC*. TGF-β signaling (19 genes): *ACVR1C, BAMBI, BMP2, CDKN2B, ID4, INHBA, LTBP1, MYC, RBL2, ROCK1, RPS6KB1, SMAD5, TGFB1, TGFB2, TGFB3, TGFBR1, TGFBR2, THBS1, TNF*. Wnt signaling (29 genes): *APC, AXIN1, BAMBI, CCND1, CCND2, CCND3, CTNNB1, FOSL1, FZD8, FZD9, GPC4, MAP3K7, MAPK10, MMP7, MYC, NFATC2, PRKACB, PRKCA, SFRP1, SFRP4, SOX11, SOX2, TP53, WNT10A, WNT11, WNT2B, WNT5A, WNT5B, WNT7B*.

**Whole-Exome Sequencing and Exome Analysis Pipeline**

Whole-exome sequencing (WES) was performed to evaluate tumor mutation burden (TMB) in 41 and 44 tumor samples obtained from the chemo-cohort and ICI combo-cohort, respectively. TMB was broadly defined as the total number of SNVs (both synonymous and nonsynonymous) and indels per tumor genomic region analyzed. DNA was extracted from FFPE tumor specimens with the use of an AllPrep DNA/RNA FFPE Kit (Qiagen), and its quality and quantity were determined with the use of a NanoDrop 2000 device (Thermo Fisher Scientific) and PicoGreen dsDNA Assay Kit (Thermo Fisher Scientific). Integrity was assessed on the basis of the DNA Integrity Number (DIN) measured with the Agilent 2200 TapeStation system (Agilent Technologies). Samples for which the concentration of extracted DNA was <10 ng/µL or the DIN was <4.5 were excluded from the analysis. Whole-exome capture libraries were constructed with the use of an Agilent Sure-Select Human All Exon v7.0 system (Agilent Technologies), with the capture region being 48.2 Mb. Samples with a library concentration of >20 ng/µL were used for further analysis. Enriched exome libraries were sequenced with the NovaSeq 6000 platform (Illumina), yielding an average of 68 million reads (10 Gb). Somatic mutations were identified with SAMtools^12^ and the Ensemble Variant Effect Predictor (VEP) pipeline.^13^ In brief, the *identify-and-annotate-variants* workflow was applied for mapping to the reference genome (hg38), identification of variants, and annotation. The *filter-somatic-variants* and *remove-variants-outside-genome-regions* workflows were then applied to remove variants outside the target (coding) regions and common variants present in publicly available databases. Sorting Tolerant From Intolerant (SIFT)^14, 15^ and Polymorphism Phenotyping v2 (PolyPhen-2) scores were obtained from VEP.^13^ Filtered mutations were evaluated as deleterious if denoted as such by both SIFT (“deleterious”) and PolyPhen-2 (“damaging”) or VEP IMPACT (high or low). SNV and indel mutation counts were computed per case. We performed the same analysis for our SCLC cohort and the lung adenocarcinoma (LUAD) data set from The Cancer Genome Atlas (TCGA).

**Neoantigen Prediction Workflow**

Human leukocyte antigen (HLA) genotypes were determined from the WES data for each patient with the use of HLAscan.^16^ HLAscan identified the four-digit HLA type as well as mutations in HLA class I genes for each sample. Mutant peptides were computed on the basis of the nsSNV and indel mutations with the use of SeqTailor.^17^ Peptide-MHC binding affinities were predicted with the use of NetMHCpan (version 4.0).^18^ Representative antigens with a binding affinity of <50 nM for HLA-A, -B, or -C were considered neoantigens.^8, 19^ We performed the same analysis for our SCLC cohort and the LUAD data set of TCGA.

**TCGA Molecular Data**

Somatic alteration data for 20 LUAD samples (top 10 and bottom 10 TMB samples) were obtained through TCGA Genomic Data Commons Data Portal as of July 2021. These samples were analyzed for TMB and predicted neoantigen burden by the same bioinformatics pipelines as those applied for our SCLC cohort.

**References** {I HAVE NOT CHECKED THESE}

1. Travis WD, Brambilla E, Nicholson AG, et al. The 2015 World Health Organization Classification of Lung Tumors: Impact of Genetic, Clinical and Radiologic Advances Since the 2004 Classification. *J Thorac Oncol* 2015;10:1243-1260.

2. Borghaei H, Paz-Ares L, Horn L, et al. Nivolumab versus Docetaxel in Advanced Nonsquamous Non-Small-Cell Lung Cancer. *N Engl J Med* 2015;373:1627-1639.

3. Haratani K, Hayashi H, Tanaka T, et al. Tumor immune microenvironment and nivolumab efficacy in EGFR mutation-positive non-small-cell lung cancer based on T790M status after disease progression during EGFR-TKI treatment. *Ann Oncol* 2017;28:1532-1539.

4. Kulangara K, Zhang N, Corigliano E, et al. Clinical Utility of the Combined Positive Score for Programmed Death Ligand-1 Expression and the Approval of Pembrolizumab for Treatment of Gastric Cancer. *Arch Pathol Lab Med* 2019;143:330-337.

5. Ott PA, Bang YJ, Piha-Paul SA, et al. T-Cell-Inflamed Gene-Expression Profile, Programmed Death Ligand 1 Expression, and Tumor Mutational Burden Predict Efficacy in Patients Treated With Pembrolizumab Across 20 Cancers: KEYNOTE-028. *J Clin Oncol* 2019;37:318-327.

6. Haratani K, Hayashi H, Takahama T, et al. Clinical and immune profiling for cancer of unknown primary site. *J Immunother Cancer* 2019;7:251.

7. Ayers M, Lunceford J, Nebozhyn M, et al. IFN-gamma-related mRNA profile predicts clinical response to PD-1 blockade. *J Clin Invest* 2017;127:2930-2940.

8. Cristescu R, Mogg R, Ayers M, et al. Pan-tumor genomic biomarkers for PD-1 checkpoint blockade-based immunotherapy. *Science* 2018;362.

9. Tomfohr J, Lu J, Kepler TB. Pathway level analysis of gene expression using singular value decomposition. *BMC Bioinformatics* 2005;6:225.

10. Danaher P, Warren S, Lu R, et al. Pan-cancer adaptive immune resistance as defined by the Tumor Inflammation Signature (TIS): results from The Cancer Genome Atlas (TCGA). *J Immunother Cancer* 2018;6:63.

11. Bindea G, Mlecnik B, Tosolini M, et al. Spatiotemporal dynamics of intratumoral immune cells reveal the immune landscape in human cancer. *Immunity* 2013;39:782-795.

12. Danecek P, Bonfield JK, Liddle J, et al. Twelve years of SAMtools and BCFtools. *Gigascience* 2021;10.

13. McLaren W, Gil L, Hunt SE, et al. The Ensembl Variant Effect Predictor. *Genome Biol* 2016;17:122.

14. Kumar P, Henikoff S, Ng PC. Predicting the effects of coding non-synonymous variants on protein function using the SIFT algorithm. *Nature Protocols* 2009;4:1073-1081.

15. Ng PC, Henikoff S. SIFT: predicting amino acid changes that affect protein function. *Nucleic Acids Research* 2003;31:3812-3814.

16. Ka S, Lee S, Hong J, et al. HLAscan: genotyping of the HLA region using next-generation sequencing data. *BMC Bioinformatics* 2017;18:258.

17. Zhang P, Boisson B, Stenson PD, et al. SeqTailor: a user-friendly webserver for the extraction of DNA or protein sequences from next-generation sequencing data. *Nucleic Acids Res* 2019;47:W623-W631.

18. Jurtz V, Paul S, Andreatta M, et al. NetMHCpan-4.0: Improved Peptide-MHC Class I Interaction Predictions Integrating Eluted Ligand and Peptide Binding Affinity Data. *J Immunol* 2017;199:3360-3368.

19. Turajlic S, Litchfield K, Xu H, et al. Insertion-and-deletion-derived tumour-specific neoantigens and the immunogenic phenotype: a pan-cancer analysis. *The Lancet Oncology* 2017;18:1009-1021.
