# Supplementary material for: The Tumor Immune Microenvironment and Frameshift Neoantigen Load Determine Response to PD-L1 Blockade in Extensive-Stage SCLC
Source: JTO Clin Res Rep. 2022 Jul 1;3(8):100373. doi: 10.1016/j.jtocrr.2022.100373 (PMC9356091; doi:10.1016/j.jtocrr.2022.100373)
Supplement: Supplementary Table S1 [file mmc7.docx]

**Supplementary Table S1. Characteristics of the Study Patients in the ICI Combo-Cohort According to Tumor Inflammation Status**

| **Characteristic** | **Number of patients (%)^a^** | | ***P* value^b^** |
| --- | --- | --- | --- |
|  | **Noninflamed tumors**  **(n=56)** | **Inflamed tumors**  **(n=7)** |  |
| Median age (range), years^c^ | 72 (34–83) | 66 (57–82) | 0.369 |
| Sex |  |  |  |
| Male | 46 (82.1) | 7 (100.0) | 0.59 |
| Female | 10 (17.9) | 0 (0) |  |
| ECOG performance status |  |  |  |
| 0–1 | 47 (83.9) | 7 (100.0) | 0.886 |
| 2 | 5 (8.9) | 0 (0) |  |
| 3–4 | 4 (7.1) | 0 (0) |  |
| Smoking status^d^ |  |  |  |
| Current or former | 56 (100.0) | 7 (100.0) | 1.00 |
| Never | 0 (0) | 0 (0) |  |
| Unknown | 0 (0) | 0 (0) |  |
| Stage |  |  |  |
| Limited | 3 (5.4) | 0 (0) | 1.00 |
| Extensive | 53 (94.6) | 7 (100.0) |  |
| Metastasis at baseline |  |  |  |
| CNS | 17 (30.4) | 3 (42.9) | 0.67 |
| Intrathoracic only | 7 (12.5) | 2 (28.6) | 0.260 |
| Extrathoracic | 46 (82.1) | 5 (71.4) | 0.610 |
| Histology |  |  |  |
| Small cell | 55 (98.2) | 5 (71.4) | 0.03 |
| Combined | 1 (1.8) | 2 (28.6) |  |
| Treatment |  |  |  |
| Surgery | 4 (7.1) | 0 (0) | 1.00 |
| Radiotherapy | 6 (10.7) | 0 (0.0) | 1.00 |
| Median (range) serum LDH, U/L | 257 (145–2143) | 242 (8.4–5860) | 0.76 |
| Median (range) serum albumin, g/dL | 3.5 (2.3–4.3) | 3.8 (3.3–4.1) | 0.24 |

Abbreviations: ECOG, Eastern Cooperative Oncology Group; CNS, central nervous system; TPS, tumor proportion score; CPS, combined positive score; NLR, neutrophil-to-lymphocyte ratio; LDH, lactate dehydrogenase.

^a^Percentages may not add up to 100 because of rounding.

^b^*P* values were determined with the Wilcoxon rank sum test or Fisher’s exact test as appropriate.

^c^At the start of treatment.

^d^Current smokers, individuals who had smoked a cigarette within the previous year; former smokers, those who had smoked ≥100 cigarettes but had quit >1 year before diagnosis; never-smokers, those who had smoked <100 cigarettes.
