# Supplementary material for: The Tumor Immune Microenvironment and Frameshift Neoantigen Load Determine Response to PD-L1 Blockade in Extensive-Stage SCLC
Source: JTO Clin Res Rep. 2022 Jul 1;3(8):100373. doi: 10.1016/j.jtocrr.2022.100373 (PMC9356091; doi:10.1016/j.jtocrr.2022.100373)
Supplement: Supplementary Table S2 [file mmc8.docx]

**Supplementary Table S2. Characteristics of the Study Patients in the Chemo-Cohort According to Tumor Inflammation Status**

| **Characteristic** | **Number of patients (%)^a^** | | ***P* value^b^** |
| --- | --- | --- | --- |
|  | **Noninflamed tumors**  **(n=57)** | **Inflamed tumors**  **(n=13)** |  |
| Median age (range), years^c^ | 73 (35–84) | 70 (61–81) | 0.839 |
| Sex |  |  |  |
| Male | 45 (79.0) | 9 (69.2) | 0.476 |
| Female | 12 (21.1) | 4 (30.8) |  |
| ECOG performance status |  |  |  |
| 0–1 | 45 (78.9) | 8 (61.5) | 0.473 |
| 2 | 8 (14.0) | 4 (30.8) |  |
| 3–4 | 4 (7.0) | 1 (7.7) |  |
| Smoking status^d^ |  |  |  |
| Current or former | 55 (96.5) | 12 (92.3) | 0.36 |
| Never | 1 (1.8) | 1 (7.7) |  |
| Unknown | 1 (1.8) | 0 (0) |  |
| Stage |  |  |  |
| Limited | 7 (12.3) | 1 (7.7) | 1.00 |
| Extensive | 50 (87.7) | 12 (92.3) |  |
| Metastasis at baseline |  |  |  |
| CNS | 9 (15.8) | 7 (53.8) | 0.007 |
| Intrathoracic only | 9 (15.8) | 0 (0) | 0.193 |
| Extrathoracic | 41 (71.9) | 12 (92.3) | 0.165 |
| Histology |  |  |  |
| Small cell | 53 (93.0) | 12 (92.3) | 1.00 |
| Combined | 4 (7.0) | 1 (7.7) |  |
| Treatment |  |  |  |
| Surgery | 7 (12.3) | 2 (15.4) | 0.67 |
| Radiotherapy | 4 (7.0) | 0 (0.0) | 1.00 |
| Median (range) serum LDH, U/L | 267 (133–998) | 321 (212–1164) | 0.334 |
| Median (range) serum albumin, g/dL | 3.7 (1.8–4.5) | 3.4 (3.0–4.5) | 0.344 |

Abbreviations: ECOG, Eastern Cooperative Oncology Group; CNS, central nervous system; TPS, tumor proportion score; CPS, combined positive score; NLR, neutrophil-to-lymphocyte ratio; LDH, lactate dehydrogenase.

^a^Percentages may not add up to 100 because of rounding.

^b^*P* values were determined with the Wilcoxon rank sum test or Fisher’s exact test as appropriate.

^c^At the start of treatment.

^d^Current smokers, individuals who had smoked a cigarette within the previous year; former smokers, those who had smoked ≥100 cigarettes but had quit >1 year before diagnosis; never-smokers, those who had smoked <100 cigarettes.
